# Is There Any Association Between Neurodegenerative Diseases and Periodontitis? A Systematic Review

**DOI:** 10.3389/fnagi.2021.651437

**Published:** 2021-05-24

**Authors:** María Olimpia Paz Alvarenga, Deborah Ribeiro Frazão, Isabella Gomes de Matos, Leonardo Oliveira Bittencourt, Nathália Carolina Fernandes Fagundes, Cassiano Kuchenbecker Rösing, Lucianne Cople Maia, Rafael Rodrigues Lima

**Affiliations:** ^1^Laboratory of Functional and Structural Biology, Institute of Biological Sciences, Federal University of Pará, Belém, Brazil; ^2^Faculty of Medicine and Dentistry, School of Dentistry, University of Alberta, Edmonton, AB, Canada; ^3^Department of Periodontology, School of Dentistry, Federal University of Rio Grande Do Sul (UFRGS), Porto Alegre, Brazil; ^4^Department of Pediatric Dentistry and Orthodontics, School of Dentistry, Federal University of Rio de Janeiro, Rio de Janeiro, Brazil

**Keywords:** neurodegenerative diseases, Alzheimer's disease, Parkinson's disease, periodontitis, systematic review

## Abstract

**Background:** Neurodegenerative diseases are a group of progressive disorders that affect the central nervous system (CNS) such as Alzheimer, Parkinson, and multiple sclerosis. Inflammation plays a critical role in the onset and progression of these injuries. Periodontitis is considered an inflammatory disease caused by oral biofilms around the tooth-supporting tissues, leading to a systemic and chronic inflammatory condition. Thus, this systematic review aimed to search for evidence in the association between neurodegenerative disorders and periodontitis.

**Methods:** This systematic review was registered at International Prospective Register of Systematic Reviews (PROSPERO) under the code CRD 42016038327. The search strategy was performed in three electronic databases and one gray literature source—PubMed, Scopus, Web of Science, and OpenGrey, based on the PECO acronym: observational studies in humans (P) in which a neurodegenerative disease was present (E) or absent (C) to observe an association with periodontitis (O). The Fowkes and Fulton checklist was used to critically appraise the methodological quality and the risk of bias of individual studies. The quality of evidence was assessed by the Grading of Recommendations Assessment, Development and Evaluation (GRADE).

**Results:** From 534 articles found, 12 were included, of which eight were case–control, three were cross-sectional, and one was a cohort, giving a total of 3,460 participants. All the included studies reported an association between some neurodegenerative diseases and periodontitis and presented a low risk of bias. According to the GRADE approach, the level of evidence of probing pocket depth was considered very low due to the significant heterogeneity across the studies' upgrading imprecision and inconsistency.

**Conclusions:** Although all the included studies in this review reported an association between neurodegenerative diseases and periodontitis, the level of evidence was classified to be very low, which suggests a cautious interpretation of the results.

## Introduction

Neurodegenerative disease is a broad expression for a group of disorders that damage the central nervous system (CNS), characterized by the progressive loss of neuronal structure and function. These diseases are incurable and lead to a progressive decline or even the complete loss of sensory, motor, and cognitive functions (Hussain et al., 2018). According to the World Health Organization (WHO), neurodegenerative disorders affect up to 1 billion people worldwide, and the proportion is growing with the aging of the world population; they lead to the death of about 6.8 million people per year, equivalent to 12% of all deaths in the world (Feigin et al., 2020).

Among the different types of neurodegenerative diseases, Alzheimer's disease (AD), Huntington's disease, Parkinson's disease (PD), and multiple sclerosis are the most frequently occurring (Hussain et al., 2018). These disorders damage the CNS and trigger rapid microglial activation, the main component of neuroinflammation. Activated microglia produce and secrete inflammatory mediators, such as eicosanoids, cytokines, chemokines, reactive free radicals, and proteases. Although a well-regulated inflammatory process is beneficial for injured CNS tissue, an excessive inflammatory response can be a source of additional injury and may affect the chronic progression of these diseases (Gao and Hong, 2008).

Some inflammatory diseases, such as periodontitis, might represent a factor that can contribute to CNS damage. Periodontitis is a multifactorial chronic inflammatory disease that affects the supporting tissues around the teeth such as gum, cementum, periodontal ligament, and alveolar bone triggered by dysbiotic biofilms that can lead to a systemic inflammatory (Papapanou et al., 2018). Periodontal disease is one of the most frequent causes of tooth loss, leading to alterations in the masticatory and aesthetic functions and, finally, impairing the quality of life of individuals (Papapanou et al., 2018). It is highly prevalent in adults affecting about 20–50% of the global population (Nazir, 2017). It can lead to a systemic inflammatory state through mechanisms that include the spread of pro-inflammatory cytokines and/or bacteria located in the oral cavity (Hajishengallis, 2015). Persistent systemic inflammation/infection can cause neuroinflammation in the brain (Perry et al., 2003).

Considering this possible interaction, the present study aims to systematically review the evidence supporting the association between the presence of some neurodegenerative disease and periodontitis.

## Methods

### Protocol and Registration

This review was conducted following the Preferred Reporting Items for Systematic Reviews and Meta-Analyses (PRISMA) (Moher et al., 2009). It was registered under number CRD: 42016038327 in the International Prospective Register of Systematic Reviews (PROSPERO) (http://www.crd.york.ac.uk).

### Search Strategy

The search strategy was performed on four electronic databases, PubMed, Scopus, Web of Science, and The Cochrane Library, and a gray literature source, OpenGrey. Both MeSH and entry terms were adapted adequately according to each database's syntax rule, as shown in Supplementary Table 1, using the operator's Booleans (OR, AND) to combine searches. The articles found in more than one database were considered only once. No restrictions were placed on publication date or language. We performed a manual search of the reference lists of the included studies to find additional articles and an alert on each database platform to detect articles on the topic published until December 2020.

### Selection and Eligibility Criteria

Studies were selected based on the PECO acronym, including observational studies in humans (P, population) in which a neurodegenerative disease was present (E, exposure) or absent (C, comparison) to observe an association between this and periodontitis (O, outcome). The aim was to answer the focused question: Is there any association between neurodegenerative disease and periodontitis in adult patients?

All titles, abstracts, and full-text reading of the articles were independently analyzed by two reviewers (MA and IM) who imported all relevant citations into a bibliographic reference manager (EndNote®, version X7, Thomson Reuters, Philadelphia, USA). In case of disagreement between the examiners, a third reviewer (RL) was involved. Studies that included patients without a diagnostic of neurodegenerative disease, groups with gingivitis only, case reports, descriptive studies, review articles, opinion articles, technical articles, guidelines, animal and *in vitro* studies were excluded.

### Data Extraction and Risk of Bias Assessment

The following data were extracted from the articles: authors and year; study design; characteristics of the sample (size, age, location, and study group); evaluation method (clinical and laboratory parameters); statistical analysis; results (study group, control group); and the outcome. Data were extracted and tabulated independently by two reviewers (MA and LB).

The checklist developed by Fowkes and Fulton (1991) was used for critical appraisal of the methodological quality and risk of bias of individual studies. This checklist has domains related to study and sample design; control group characteristics; quality of measures and results; integrity; and distorted influences. For each criterion, a sign was assigned (++) in case of major problems in the study or (+) in case of minor issues, to assess whether the methods are adequate to produce consistent and valid information, as well as whether the results offered the expected effects that might infer conclusions. In areas where the question did not apply to the type of study, NA was assigned (not applicable). No problem has been designated by the sign (0). The evaluation for each domain was standardized by the examiners, as described in Supplementary Table 2. After assessment of each field, the studies were analyzed to determine the value of the study through three summary questions: “Are the results erroneously biased in a certain direction?”; “Are there any serious confounding or other distorting influences?”; and “Is it likely that the results occurred by chance?”. These items were assigned “yes” and “no” answers. If the answer is no, in the three questions, the article is considered reliable, with a low risk of bias.

### Level of Evidence (Grading of Recommendations Assessment, Development, and Evaluation)

The quality of evidence was rated using the GRADE approach (Movsisyan et al., 2016). A narrative assessment was chosen according to the types of studies selected by eligibility. According to the GRADE parameters, when observational studies are considered, a “low” rating is initially given. Then, assessments within the magnitude of the effect, dose–response relationship in development, and counteracting plausible residual bias or confounding may be used to upgrade the initial “low” rating. However, if there are serious or very serious issues related to the risk of bias, inconsistency, indirectness, imprecision, and publication bias, the level of evidence declines to “very low.” Thus, the certainty of evidence can be categorized into 1 of 4 ratings—high, moderate, low, and very low—reflecting the extent to which the review authors are confident that an estimate of the effect for a specific outcome is correct (Movsisyan, 2018).

## Results

### Study Selection

**Figure 1:**
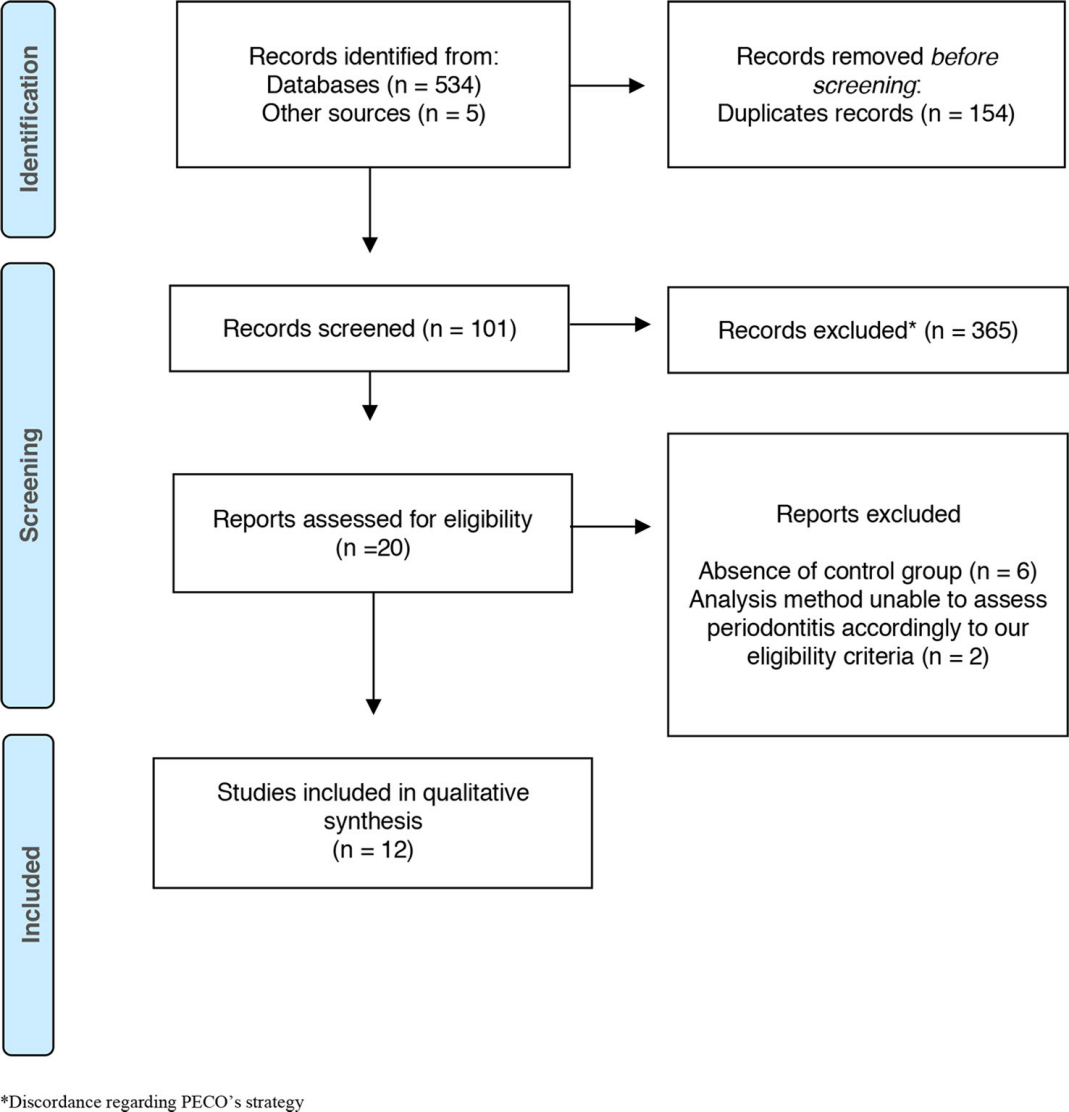
Preferred Reporting Items for Systematic Reviews and Meta-Analyses (PRISMA) flow diagram of the different phases for exclusion and inclusion of the studies. *Discordance PECO's strategy.

We identified 534 articles in the databases and the gray literature source accessed using our search strategy. After the duplicates were removed automatically and manually, 385 citations were screened by title and abstract, and 284 were excluded because they did not meet our eligibility criteria. In this phase, 101 citations were analyzed for potential eligibility, but only 20 studies remained for full-text reading from which eight articles were excluded because they did not meet the inclusion criteria (Supplementary Table 3). Finally, 12 studies were considered for qualitative analysis. All the selection phases are described in a flow diagram in Figure 1.

### Characteristics of the Included Studies

All of the included articles were observational studies: two cross-sectional, eight case–control, and two retrospective cohorts. The age range comprises 40–90 years and was considered a confounding factor and controlled in all the analyses. All studies evaluated the association between neurodegenerative disease and clinical and/or laboratory parameters of periodontitis, as described in Table 1.

**Table 1:** Characteristics of the included studies.

**Author/year/country/study design**	**Sample**			**Methods**			**Statistical analyses**	**Results**
	**Study group**	**Size**	**Mean age (years)**	**Periodontal parameters**	**Laboratory**	**Cognitive assessment**		
Aragón et al. (2018) Spain Cc	AD	*N* = 106 AD = 70 CG = 36	AD: 77.4 ± 10.6 CG: 62.6 ± 7.1	CPI	–	MMSE; Mini-Cog Test; the clock draw test; FAST; GDS.	Chi-square, test, linear regression	The CPI assessment showed worse periodontal status in AD patients than controls (0.1 ± 0.4 vs. 1.4 ± 2.2).
Holmer et al. (2018) Sweden Cc	AD	*N* = 128 AD = 52 CG = 76	AD: 71 CG: 67	PPD, %BOP, suppuration, tooth mobility, and furcation involvement.	—	MMSE	Chi-squared test or Fisher's exact test and binary logistic regression	Clinical periodontal parameters assessed were significantly worse in patients with AD than controls. The cases group was associated with generalized marginal alveolar bone loss (OR = 5.81; 95% CI = 1.14–29.68), increased number of deep periodontal pockets (OR = 8.43; CI = 4.00–17.76).
Cestari et al. (2016) Brazil Cc	AD	*N* = 65 AD = 25 CG = 21	63–92	BI, PPD, CAL, PI	Pro-inflammatory cytokines levels (IL-1, IL-6, and TNF-α)	MMSE	ANOVA, chi-square test, MANOVA	The PI and BI were significantly higher in AD patients than controls. No significant differences were founded in PPD and CAL levels. The multivariate analysis showed an association between IL-6 and TNF-α in patients with AD and periodontitis (*p* = 0.023).
Martande et al. (2014) India Cs	AD	*N* = 118 AD = 58 CG = 60	50–80	PI, GI, %BOP, PPD, CAL.	–	MMSE	ANOVA	Individuals with AD showed higher values of periodontal parameters when compared with controls. The intergroup analysis showed that periodontal condition worsened as the disease level progressed from mild to severe. The mean MMSE score for AD was 14.2 + 8.4 vs. 28.5 + 1.2 for ND individuals.
Noble et al. (2014) United States Pc	AD	*N* = 219 AD = 110 CG = 109	65–84	–	Serum IgG levels for bacteria known to be associated with periodontitis	Modified MMSE	Multivariable Cox proportional hazards, regression models	In this community-based, multiethnic cohort of elders, serum IgG levels to common periodontal microbiota are associated with risk for developing incident AD (HR 52.0, 95% CI = 1.1–3.8).
Sheu and Lin (2013) Taiwan Cc	MS	*N* = 1896 MS = 31 CG = 1580	43.7 ± 16.3	%BOP, PPD, radiographs.	–	NHIRD	Chi-square, test, logistic regressions	Among females, MS patients were significantly associated with earlier worse clinical parameters of periodontitis. However, there was no significant association among males (adjusted OR = 2.08; 95% CI = 1.49–2.95).
Stein et al. (2012) United States Pc	AD	*N* = 112 AD = 35 CG = 77	31–70	–	Serum IgG levels to seven oral bacteria associated with periodontopathic biofilms	MMSE	General linear regression models	Elevated antibodies to periodontal disease bacteria in subjects' years before cognitive impairment and suggests that periodontal disease could potentially contribute to the risk of AD onset/progression.
Müller et al. (2011) Germany Cc	PD	*N* = 176 PD = 101 GG = 75	66.2 ± 10	PB, PI, API, CAL, OHI	–	UPDRS	Parametric tests, *t*-test.	PD patients were found to have more severe clinical parameters of periodontitis.
Syrjälä et al. (2012) Finland Cs	AD	*N* = 327 AD = 49 CG = 278	83.7 ± 4.9	PPD	–	DSM-IV	Multivariate regression models, logistic regression models	AD patients and those with other types of dementia had an increased likelihood of having teeth with worse PPD.
Hanaoka and Kashihara (2009) Japan Cc	PD	*N* = 157 PD = 89 CG = 68	72.1 ± 5.5	PPD	–	MMSE	One-way ANOVA, Bonferroni, tests *post-hoc*.	The frequency of deep periodontal pocket was higher for patients with PD compared with the control.
Kamer et al. (2009) United States Cc	AD	*N* = 34 AD = 18 CG = 16	40–80	–	Serum IgG levels for bacteria known to be associated with periodontitis	MMSE	*t*-test, Mann-Whitney	Higher IgG levels of periodontal bacteria had associated with AD (Mann–Whitney *U*-test, *p* = 0.007).
Einarsdóttir et al. (2009) Iceland Cc	PD	*N* = 122 PD = 67 CG = 55	<60 to <70	PPD, API, radiograph	–	–	*t*-test	PD patients had worse scores of API and PPD compared with controls.

*Cs, cross-sectional study; Cc, case–control study; Pc, prospective cohort study; AD, Alzheimer's disease; PD, Parkinson disease; ME, multiple sclerosis; MMSE, Mini-Mental State Examination; FAST, Functional Assessment Staging of Alzheimer's Disease; DSM-IV, Diagnostic and Statistical Manual of Mental Disorders, Fourth Edition, Revised; NHIRD, NHI Research Database; UPDRS, Unified Parkinson Disease Rating Scale; CDR, Clinical Dementia Rating; GDS, Global Deterioration Scale; CPI, Community Periodontal Index; MABL, marginal alveolar bone loss; PI, plaque index; GI, gingival index; PPD, Probing Pocket Depth; CAL, clinical attachment level; %BOP, percentage bleeding on probing; PBI, papillary bleeding index; API, approximate plaque index; OHI, oral hygiene index; IgG, immunoglobulin G; ANOVA, analysis of variance; MANOVA, multivariate analysis of variance; OR, odds ratio; CI, confidence interval*.

Out of the 12 studies, eight involved patients diagnosed with AD. The cognitive status was assessed through validated instruments, most of them using the scores of the Mini-Mental State Examination (MMSE), modified (Noble et al., 2014) or not (Kamer et al., 2009; Stein et al., 2012; Martande et al., 2014; Cestari et al., 2016; Aragón et al., 2018; Holmer et al., 2018). In one study, besides the MMSE, they used a Mini-Cog Test, the clock draw test, the Functional Assessment Staging of Alzheimer's Disease (FAST), the Clinical Dementia Rating (CDR), and the Global Deterioration Scale (GDS) (Aragón et al., 2018). One study used the *Diagnostic and Statistical Manual of Mental Disorders*, 4th Edition (DSM-IV) (Syrjälä et al., 2012).

PD patients were evaluated in three studies (Einarsdóttir et al., 2009; Hanaoka and Kashihara, 2009; Müller et al., 2011); one of them assessed the global cognitive function using the MMSE (Hanaoka and Kashihara, 2009); another used the scores of the Unified Parkinson Disease Rating Scale (UPDRS) (Müller et al., 2011); and in the last study, the instrument used is unclear (Einarsdóttir et al., 2009).

Only one included a study that evaluated patients diagnosed with multiple sclerosis. In this study, the authors assessed cognition status through the NHI Research Database (NHIRD) (Sheu and Lin, 2013).

All the studies that met eligibility criteria assessed periodontitis through at least one validated clinical parameter of periodontitis, such as clinical attachment loss (CAL) or probing depth (PPD >3 mm), bleeding on probing (BOP% >25%) of evaluated sites, and/or >30% of radiographic bone loss. Another criterion that was considered in some studies was the Community Periodontal Index (CPI) score 3 (PPD of 3.5–5.5 mm) and score 4 (PPD >5.5 mm) (Einarsdóttir et al., 2009; Hanaoka and Kashihara, 2009; Müller et al., 2011; Syrjälä et al., 2012; Sheu and Lin, 2013; Martande et al., 2014; Cestari et al., 2016; Aragón et al., 2018; Holmer et al., 2018). One study also evaluated pro-inflammatory cytokine levels (IL-1, IL-6, and TNF-α) (Cestari et al., 2016). Three studies assessed serum immunoglobulin G (Bos et al., 2015) levels of bacteria associated with periodontopathic biofilms (Kamer et al., 2009; Stein et al., 2012; Noble et al., 2014).

### Risk of Bias Assessment

In the individual assessment for risk of bias, we found minor problems in some domains. In the item sampling method, six studies have minor issues since they performed convenience samples, unspecified sampling method, invited patients, and consecutive selection. Only one study (Holmer et al., 2018) reported the calculation of sample size. Two studies (Kamer et al., 2009; Cestari et al., 2016) presented a major problem in this domain, not describing the sample size calculation and having a sample size smaller than 50. For matching/randomization, one article presented a major problem due to the absence of matching groups (Einarsdóttir et al., 2009). Only one study highlighted blinding both among evaluators and in the research subjects (Martande et al., 2014). Each study assessment is shown in Table 2.

**Table 2:** Risk of bias assessment of the included studies.

**Guideline**	**Checklist**	**Aragón et al., 2018**	**Holmer et al., 2018**	**Cestari et al., 2016**	**Noble et al., 2014**	**Martande et al., 2014**	**Sheu and Lin, 2013**	**Stein et al., 2012**	**Müller et al., 2011**	**Syrjälä et al., 2012**	**Einarsdóttir et al., 2009**	**Hanaoka and Kashihara, 2009**	**Kamer et al., 2009**
Study design appropriate to objectives?	Objective common design												
	Prevalence cross-sectional												
	Prognosis cohort												
	Treatment controlled trial												
	Cause cohort, case–control, cross-sectional	0	0	0	0	0	0	0	0	0	0	0	0
Study sample representative?	Source of sample	0	0	0	0	0	0	0	0	0	+	0	0
	Sampling method	+	0	+	+	+	+	0	0	0	+	+	+
	Sample size	0	0	+	0	0	0	0	0	0	0	0	++
	Entry criteria/exclusion	0	0	0	0	0	0	0	0	0	0	0	0
	Non-respondents	0	0	0	0	0	0	0	0	0	0	0	0
Control group acceptable?	Definition of controls	0	0	0	0	0	0	0	0	0	0	0	0
	Source of controls	0	0	0	0	0	0	0	0	0	0	0	0
	Matching/ randomization	0	0	0	0	0	0	0	0	0	+	0	0
	Comparable characteristics	0	0	0	0	0	0	0	0	0	0	0	0
Quality of measurements and outcomes?	Validity	0	0	0	+	0	0	+	0	0	+	0	+
	Reproducibility	0	0	0	0	0	0	0	0	0	+	0	+
	Blindness	++	++	++	++	0	++	++	++	++	++	++	++
	Quality control	+	+	0	+	0	0	+	0	0	+	0	+
Completeness	Compliance	0	0	0	0	0	0	0	0	0	0	0	0
	Drop outs	0	0	0	0	0	0	0	0	0	0	0	0
	Deaths	NA	NA	NA	NA	NA	NA	NA	NA	NA	NA	NA	NA
	Missing data	0	0	0	0	0	0	0	0	0	0	0	0
Distorting influences?	Extraneous treatments	0	0	0	0	0	0	0	0	0	0	0	0
	Contamination	NA	NA	NA	NA	NA	NA	NA	NA	NA	NA	NA	NA
	Changes over time	0	0	0	0	0	0	0	0	0	0	0	0
	Confounding factors	0	+	0	+	+	0	+	+	+	+	+	+
	Distortion reduced by analysis	0	0	+	0	0	0	0	+	0	+	0	0
Summary questions	**Bias:** Are the results erroneously biased in a certain direction?	NO	NO	NO	NO	NO	NO	NO	NO	NO	NO	NO	NO
	**Confounding:** Are there any serious confusing or other distorting influences?	NO	NO	NO	NO	NO	NO	NO	NO	NO	NO	NO	NO
	**Chance:** Is it likely that the results occurred by chance?	NO	NO	NO	NO	NO	NO	NO	NO	NO	NO	NO	NO

*0, no problem; +, minor issues; ++, major problems; NA, not applicable*.

However, all articles are liable to be reproduced, describing all methods used in their work and including qualified and calibrated evaluators to reduce bias. There was no sample loss in any of the included studies and nor refusal to participate—it was assumed because the sample size remained the same from beginning to end. Data were collected in the same period, and confounding factors were adjusted without interfering with the results. Despite that some domains presented minor and major problems, these issues did not influence the overall judgment; hence, we judged them to be low risk of bias.

### Level of Evidence (Grading of Recommendations Assessment, Development, and Evaluation)

In the narrative of the certainty of evidence using the GRADE tool, the clinical parameter of probing pocket depth was used. The index was divided by the association with PD and AD, which was further separated for case–control and cross-sectional studies. The certainty of the evidence was considered very low for all analyses due to their inconsistency because the methods of analysis were different, making it challenging to gather the evidence. The PPD for AD in cross-sectional studies had a serious imprecision because the separation of the exposed group and the control group was performed in different ways in the two studies, hindering the accuracy of the evidence, as shown in Table 3.

**Table 3:** Level of evidence by Grading of Recommendations Assessment, Development and Evaluation (GRADE) approach.

**Certainty assessment**							**Impact**	**Certainty**	**Importance**
**No. of studies**	**Study design**	**Risk of bias**	**Inconsistency**	**Indirectness**	**Imprecision**	**Other considerations**			
**Probing pocket depth and Parkinson disease**									
2	Observational studies	Not serious	Serious^a^	Not serious	Not serious	None	One study showed that the mean pocket depth (mm) from subjects with Parkinson's disease was higher than in the control group (4.15; 3.81; *p* < 0.05). The other study demonstrated that the frequency of deep periodontal pocket (>4 mm) was higher for patients with PD compared with the control (98.6%; 43.5%; *p* < 0.001).	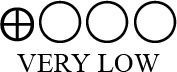	Important
**Probing pocket depth and Alzheimer's disease in case–control studies**									
2	Observational studies	Not serious	Serious^a^	Not serious	Not serious	None	One of the studies did not show a significant difference for the mean PPD on subjects with Alzheimer's disease compared with controls (2.82 ± 1.68; 2.63 ± 3.25; *p* = 0.766). The other study, however, reported that 57.7% of the diseased group had ≥9 teeth with PPD 4–5 mm, while only 23.7% of the control had this value (*p* < 0.001). Also, 71.2% of the Alzheimer group had more than 1 tooth with PPD ≥ 6, against 17% of the controls (*p* < 0.001).	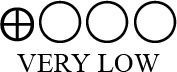	Important
**Probing pocket depth and Alzheimer's disease in cross-sectional studies**									
2	Observational studies	Not serious	Serious^a^	Not serious	Serious^b^	None	One study compared healthy individuals to individuals with mild to severe Alzheimer's levels. The mean PPD was significantly higher in the AD groups compared with the control. The other study, however, verified the mean of teeth with PPD 4 mm, reporting that there was no statistically significant difference.	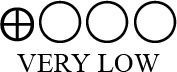	Important

a *Although the studies found similar results, their methods of analysis were different, making it difficult to gather the evidence*.

b *The separation of the exposed group and the control group was performed differently in the two studies, hindering the accuracy of the evidence*.

## Discussion

The present systematic review summarized the evidence supporting the association between periodontitis and neurodegenerative diseases, especially AD and PD. The selection of studies to be included was performed after rigid criteria. All studies presented a low risk of bias and reported an association between neurodegenerative disease and periodontitis. The articles showed that the groups with the two concomitant diseases had higher inflammatory markers levels, IgG levels of periodontal bacteria, and/or clinical parameters of periodontitis compared with the isolated conditions. However, the heterogeneity of the studies taken together hindered the accuracy of the evidence and also made impossible the merging of data. In this sense, the present systematic review sticks to a qualitative analysis of the literature.

Periodontitis is an inflammatory disease triggered by dysbiotic biofilms and the most severe form of periodontal disease (Saini et al., 2011). It is diagnosed using clinical and radiographic evaluation of periodontal parameters, such as clinical attachment loss, probing depth, bleeding on probing, and/or radiographic bone loss (Armitage, 1999; Papapanou et al., 2018). In worse cases, the radiographic analysis shows an extensive loss of the bone supporting the compromised teeth (Page and Kornman, 1997). The dental biofilm is primarily composed of several gram-negative bacteria; the most relevant is *Porphyromonas gingivalis*, which stimulates the immuno-inflammatory response of the organism (Gaur and Agnihotri, 2015), followed by *Prevotella intermedia, Fusobacterium nucleatum, Tannerella forsythia*, and *Treponema denticola* (Bartova et al., 2014; Papapanou et al., 2018). The importance of specific bacteria is continuously subject to controversy since the understanding of the dysbiosis that occurs in periodontal tissues goes beyond the particular infection.

All the included studies in this review assessed periodontal status by clinical or laboratory parameters in subjects with a neurodegenerative disease (i.e., AD, PD, or multiple sclerosis). Nine studies assessed periodontitis by clinical parameters such as CPI, CAL, BOP, and PPD (Einarsdóttir et al., 2009; Hanaoka and Kashihara, 2009; Müller et al., 2011; Syrjälä et al., 2012; Sheu and Lin, 2013; Martande et al., 2014; Cestari et al., 2016; Aragón et al., 2018; Holmer et al., 2018). Three studies only assessed the serum IgG levels of the oral bacteria associated with periodontopathic biofilms (Kamer et al., 2009; Stein et al., 2012; Noble et al., 2014); one study evaluated periodontitis by clinical parameters and also assessed pro-inflammatory cytokines levels (IL-1, IL-6, and TNF-α) in serum (Cestari et al., 2016). The immune-inflammatory response triggered by periodontal pathogens turns the individual more susceptible to several systemic diseases (Friedewald et al., 2009; Hanes and Krishna, 2010; Otomo-Corgel et al., 2012; Tonetti et al., 2013; Ferreira et al., 2017; Martelli et al., 2017; Syahputra et al., 2018) including dementia (National Academies of Sciences, 2017) and neurodegenerative diseases (Webster et al., 2008; Kamer et al., 2009).

Neurodegenerative disease is a broad term for some illnesses that progressively affect the function of neurons in the human brain such as multiple sclerosis, PD, and AD (Bertram et al., 2005). These conditions are incurable and result in progressive degeneration and/or death of neurons, triggering ataxias and dementias (Bredesen et al., 2006). It has been advocated that periodontitis is associated with neurodegenerative diseases by two pathways: the most studied relates to the fact that the mediators of inflammation are present in the blood circulation, generating a constant inflammatory status (Teixeira et al., 2017); another possibility is related to gram-negative bacteria involved, e.g., *P. gingivalis* could directly induce damage (Dominy et al., 2019).

Gram-negative bacteria present lipopolysaccharides (LPSs), B-lymphocyte activators. *P. gingivalis* presents a cysteine protease called gingipain. This protease is divided into molecules of CD14, a receptor for the LPS enzyme, which allows bacteria to suppress the immune reaction against LPS (Bainbridge and Darveau, 2001). The rupture of the periodontal pocket is an easy access route for periodontal bacteria in the systemic circulation (Curtis, 2014). As a result, pro-inflammatory cytokines that are located in this area are taken to the systemic circulation, making periodontitis no longer a local inflammation and can be considered as “low-grade systemic inflammation;” increasing the inflammatory pool in the brain by two different pathways, by systemic circulation and by neural pathways; compromising the blood–brain barrier (BBB); and having facilitated access to the brain (D'aiuto et al., 2005). The constant exchange of solutes from the fluids of soft tissues and the blood plasma allows the systemic circulation of inflammatory-related molecules, which can trespass the BBB (D'aiuto et al., 2005).

The neural pathway might be conditioned to both the pathophysiology of the neurodegenerative diseases and the increase of systemic pro-inflammatory cytokines. Microglial cells are the predominant immune cells of the brain, and preclinical evidence also suggests the association between systemic inflammation and microglial activation, which can play a detrimental role in the progression of neurodegenerative due to the neurotoxicity mediated by oxidative stress and inflammation, leading to neuronal death (Holmes, 2013; Hoogland et al., 2015). The microglia can be stimulated not only by its neural microenvironment but by systemic stimuli as well (Perry and Teeling, 2013). Some evidence has shown the involvement of toll-like receptor activation (Bilbo et al., 2010) *via* pathogen-associated molecular patterns, complement 1q, and adenosine triphosphate release from astrocytes, producing TNF-α, Il-1β, and Il-6 (Hoogland et al., 2015; Tang and Le, 2016). In this way, as previously reviewed by Teixeira et al. (2017), the cross talk between neurodegenerative diseases and periodontitis underlies the priming of the microglia, i.e., the phenotype switch, in which the cell adopts a pro-inflammatory profile, aggravating the neuroinflammation.

Out of the 12 included studies, eight evaluated the possible association between periodontitis and AD (Kamer et al., 2009; Stein et al., 2012; Syrjälä et al., 2012; Martande et al., 2014; Noble et al., 2014; Cestari et al., 2016; Aragón et al., 2018; Holmer et al., 2018). Patients with AD present cognitive and behavioral damage (Hill et al., 2014), consistent neuroinflammation with infection, microglial activation, inflammasome activation, complement activation, and altered cytokine profiles (Dominy et al., 2019). Infections or systemic peripheral inflammation has been associated with the onset and progression of cognitive decline (Cunningham and Hennessy, 2015). The expression of TNF-α is considered an essential inflammatory cytokine to regulate the cascade of cellular events that occur during the neuroinflammatory response (Gurav, 2014). This inflammatory marker is up-regulated in AD and used as a diagnostic for this disease (Kamer et al., 2009; Ding et al., 2019). In one of our included studies, the multivariate analysis showed an association between IL-6 and TNF-α in patients with AD and periodontitis (Cestari et al., 2016). The concentration of IL-6 was negatively correlated with the MMSE, meaning that subjects with low scores in the cognitive test had high serum IL-6 levels. On the other hand, TNF-α concentration was positively correlated with PPD and CAL, showing that patients with worse periodontal conditions had more elevated TNF-α levels in serum. This finding suggests that immune-inflammatory mechanisms of periodontitis may underlie its role in the onset, progression, or aggravation of AD (Cestari et al., 2016). Previous studies have reported higher levels of inflammatory molecules in the brain in the presence of periodontitis (Kamer et al., 2009), playing an essential role in neuroinflammation and a potential risk factor for the incidence and progression of AD (Wu and Nakanishi, 2014).

Another pathway in the association between periodontitis and AD is related to periodontitis's main pathogenic bacteria (Underly et al., 2016; Dominy et al., 2019). *P. gingivalis* was identified in the brain of AD patients; the bacteria levels were positively correlated with the pathology of tau and ubiquitin (Dominy et al., 2019), classical biological markers of this disease (Underly et al., 2016). Out of the eight studies evaluating this association, three evaluated the serum IgG levels for bacteria associated with periodontitis. They detected higher levels of antibodies in patients diagnosed with the two concomitant diseases (Kamer et al., 2009; Stein et al., 2012; Noble et al., 2014). In the five remaining studies, the authors reported worse scores in at least one clinical parameter of periodontitis in patients with AD (Syrjälä et al., 2012; Martande et al., 2014; Cestari et al., 2016; Aragón et al., 2018; Holmer et al., 2018), and the intergroup analysis in one of them showed that periodontal condition worsened as the disease level presented higher severity (Martande et al., 2014).

PD is another neurodegenerative disease associated with periodontitis. This disease is related to movement disorders, degeneration of dopaminergic neurons, and the presence of cytoplasmic inclusion bodies—known as Lewy bodies (Matsui and Takahashi, 2018). PD is the second most common progressive neurodegenerative disease after AD (Kaur et al., 2016). Similar to the mechanism in AD, the bacteremia and systemic translocation triggered by the gram-negative bacteria present in periodontitis (Ebersole and Cappelli, 2000) might initiate and progress PD (Kaur et al., 2016). The rupture of the BBB allows the entry of macrophages and pro-inflammatory mediators, turning inactive microglia into active ones. When activated, they can produce several inflammatory mediators, such as TNF-α, Il-1β, Il-6, iNOS, and reactive oxygen species (ROS), triggering necrosis and, finally, apoptosis of dopaminergic neurons in the CNS, marking the onset and/or progression of PD. Three of the 12 studies included evaluated this association and detected patients with PD having more severe clinical parameters of periodontitis (Einarsdóttir et al., 2009; Hanaoka and Kashihara, 2009; Müller et al., 2011).

Multiple sclerosis is not yet fully understood, but multiple infections are crucial in the development of the disease; thus, it has also been associated with bacteremia present in periodontitis (Sheu and Lin, 2013). This neurodegenerative disease is an inflammatory condition characterized by demyelination and axonal degeneration. Multiple sclerosis is considered an autoimmune-mediated disease, resulting from several interactions between environmental factors and genetic predisposition (Sheu and Lin, 2013; Bos et al., 2015). These interactions cause an autoimmune response, which in turn causes inflammation in the myelin sheath that surrounds the axons of the CNS (Sheu and Lin, 2013). This disease caused more burden in women than in men (Feigin et al., 2020). One study in our review evaluated this association and conducted further analysis on the odds ratio for earlier worse clinical parameters of periodontitis by sex and detected higher odds in female patients only (Sheu and Lin, 2013); this finding might be related to gender differences in immune responses to chronic infection.

Regarding the methodological quality and risk of bias of individual studies, the checklist developed by Fowkes and Fulton was used (Fowkes and Fulton, 1991), which evaluates the articles according to sample design, control group characteristics, quality of measures and results, integrity, and confounding factors. The main problems were related to the sampling method, sample calculation, and the definition of the control group. Despite that, all included articles were disposed to reproducibility, because they had careful evaluators and calibration to reduce bias.

However, even though they had a low risk of bias, the certainty of the evidence for all analyzed parameters was considered as very low according to the GRADE approach. For this systematic review, it was possible only to gather sufficient information about probing pocket depth for the analysis of the level of evidence. Even though the outcomes presented serious problems related to inconsistency and imprecision because there is high heterogeneity across the included studies, the forms of analysis of the results were different from each study. Also, the separation of the exposed group and the control group was performed differently, hindering the accuracy of the evidence.

## Limitations

The main limitation of performing this systematic review was the impossibility of merging data to perform a quantitative analysis. The studies present high heterogeneity due to the variations in the assessment of periodontitis and neurodegenerative across them. Also, it should be highlighted that no cohort study was retrieved regarding the association between periodontal disease and neurodegenerative diseases; therefore, causality cannot be claimed. On the other hand, associations found that inadequately performed studies allow hypotheses to be raised regarding risk. Such approaches can be further studied both to establish a risk factor and to look for common risk factors between both diseases that should be commonly targeted. Also, a bidirectional interference could be considered since the potentially impaired dexterity of arms and fingers in PD, for example, may interfere in oral hygiene care (Vanbellingen et al., 2011).

## Conclusions

**Figure 2:**
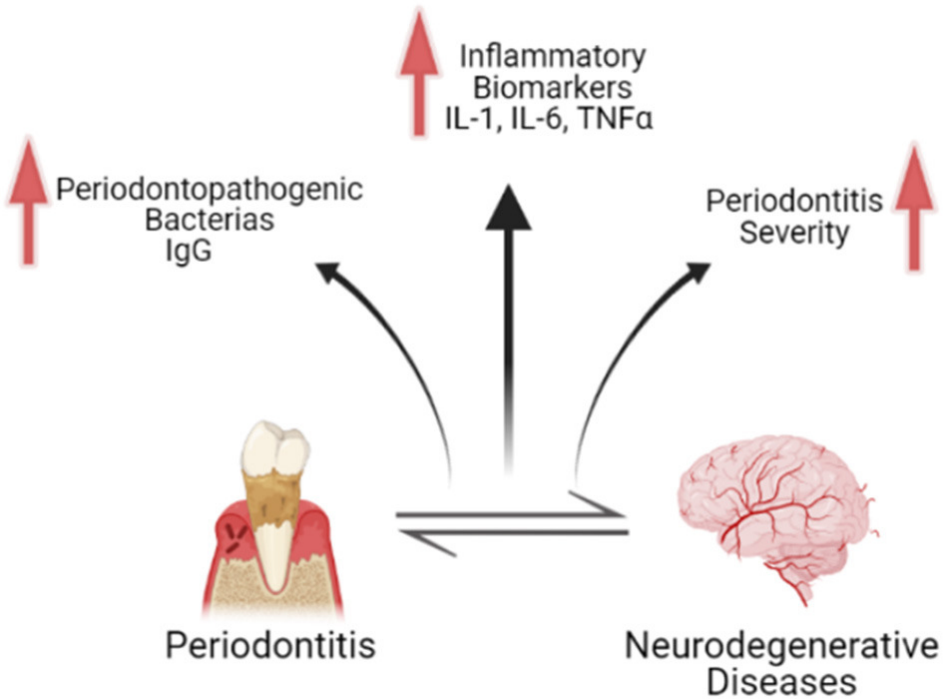
Schematic figure representing the main findings regarding the association between periodontitis and neurodegenerative diseases. The bidirectional relationship between both conditions is associated with the increase of inflammatory biomarkers, increase in immunoglobulins G related to periodontopathogenic bacteria, and increase in periodontitis severity. Schematic figure created with biorender.com.

All the included studies reported higher levels of inflammatory markers, IgG levels of periodontal bacteria, and/or clinical parameters of periodontitis with the two concomitant diseases (some neurodegenerative disease and periodontitis), compared with the diseases isolated (summarized in Figure 2). Despite that we found some minor problems in domains in the qualitative analysis, all the studies have consistent and valid information and were judged to be at low risk of bias. However, when we analyzed the body of the evidence, some problems regarding the imprecision and inconsistency hindered the accuracy of the evidence, alerting us to interpret cautiously the results.

More longitudinal studies and multicenter trials with larger sample sizes should be conducted to assess whether periodontitis could be a risk factor for the onset and/or progression of neurodegenerative diseases, impacting the quality of life in elderly people. At the moment, it can be concluded that there is an association between neurodegenerative diseases and periodontitis, but causality cannot be claimed.

## Data Availability Statement

The original contributions presented in the study are included in the article/Supplementary Materials, further inquiries can be directed to the corresponding author.

## Author Contributions

MA drafted the paper with input from all authors. NF and LM designed the study. MA, IM, and LB performed the searches and data extraction. MA and DF performed and interpreted the qualitative analysis. RL and CR revised the manuscript critically for important intellectual content and final approval of the version to be published.

## Conflict of Interest

The authors declare that the research was conducted in the absence of any commercial or financial relationships that could be construed as a potential conflict of interest.
