# Supplementary material for: Is There Any Association Between Neurodegenerative Diseases and Periodontitis? A Systematic Review
Source: Front Aging Neurosci. 2021 May 24;13:651437. doi: 10.3389/fnagi.2021.651437 (PMC8180549; doi:10.3389/fnagi.2021.651437)
Supplement: Supplementary file 1 [file Table_1.DOCX]

**Supplementary Table 1.** Search strategy for each database.

| **Databases** | **Combined** |
| --- | --- |
| PUBMED | (((((((((((((((((((((((((((((((((((("Chronic Periodontitis"[MeSH Terms]) OR "Chronic Periodontitis"[Title/Abstract]) OR "Periodontitis, Adult"[Title/Abstract]) OR "Chronic Periodontitides"[Title/Abstract]) OR "Periodontitides, Chronic"[Title/Abstract]) OR "Periodontitis, Chronic"[Title/Abstract]) OR "Adult Periodontitis"[Title/Abstract]) OR "Adult Periodontitides"[Title/Abstract]) OR "Periodontitides, Adult"[Title/Abstract]) OR "Periodontal Atrophy"[Title/Abstract]) OR "Gingivo-Osseous Atrophies"[Title/Abstract]) OR "Periodontal Atrophies"[Title/Abstract]) OR "Atrophy of Periodontium"[Title/Abstract]) OR "Periodontium Atrophies"[Title/Abstract]) OR "Periodontium Atrophy"[Title/Abstract]) OR "Gingivo-Osseous Atrophy"[Title/Abstract]) OR "Gingivo Osseous Atrophy"[Title/Abstract]) OR "Alveolar Bone Loss"[Title/Abstract]) OR "Bone Loss, Alveolar"[Title/Abstract]) OR "Alveolar Bone Losses"[Title/Abstract]) OR "Alveolar Process Atrophy"[Title/Abstract]) OR "Alveolar Process Atrophies"[Title/Abstract]) OR "Alveolar Resorption"[Title/Abstract]) OR "Alveolar Resorptions"[Title/Abstract]) OR "Resorption, Alveolar"[Title/Abstract]) OR "Resorptions, Alveolar"[Title/Abstract]) OR "Bone Loss, Periodontal"[Title/Abstract]) OR "Bone Losses, Periodontal"[Title/Abstract]) OR "Periodontal Bone Losses"[Title/Abstract]) OR "Periodontal Bone Loss"[Title/Abstract]) OR "Periodontal Resorption"[Title/Abstract]) OR "Periodontal Resorptions"[Title/Abstract]) OR "Resorption, Periodontal"[Title/Abstract]) OR "Alveolar Bone Atrophy"[Title/Abstract]) OR "Alveolar Bone Atrophies"[Title/Abstract]) OR "Bone Atrophies, Alveolar"[Title/Abstract]) OR "Bone Atrophy, Alveolar"[Title/Abstract] AND  ((((((((((((((((((((((((("Motor Neuron Disease"[MeSH Terms]) OR "Motor Neuron Disease"[Title/Abstract]) OR "Primary Lateral Scleroses"[Title/Abstract]) OR "Motor Neuron Diseases"[Title/Abstract]) OR "Neuron Disease, Motor"[Title/Abstract]) OR "Neuron Diseases, Motor"[Title/Abstract]) OR "Motor System Disease"[Title/Abstract]) OR "Motor System Diseases"[Title/Abstract]) OR "Familial Motor Neuron Disease"[Title/Abstract]) OR "Motor Neuron Disease, Familial"[Title/Abstract]) OR "Lateral Sclerosis"[Title/Abstract]) OR "Lateral Scleroses"[Title/Abstract]) OR "Scleroses, Lateral"[Title/Abstract]) OR "Sclerosis, Lateral"[Title/Abstract]) OR "Primary Lateral Sclerosis"[Title/Abstract]) OR "Lateral Scleroses, Primary"[Title/Abstract]) OR "Lateral Sclerosis, Primary"[Title/Abstract]) OR "Lower Motor Neuron Disease"[Title/Abstract]) OR "Scleroses, Primary Lateral"[Title/Abstract]) OR "Sclerosis, Primary Lateral"[Title/Abstract]) OR "Motor Neuron Disease, Upper"[Title/Abstract]) OR "Upper Motor Neuron Disease"[Title/Abstract]) OR "Motor Neuron Disease, Secondary"[Title/Abstract]) OR "Secondary Motor Neuron Disease"[Title/Abstract]) OR "Anterior Horn Cell Disease"[Title/Abstract]) OR "Motor Neuron Disease, Lower"[Title/Abstract] OR (((((((((((((((((((((((((((((((((((((((("Creutzfeldt-Jakob Syndrome"[MeSH Terms]) OR "Creutzfeldt-Jakob Syndrome"[Title/Abstract]) OR "Disease, Creutzfeldt Jacob"[Title/Abstract]) OR "Creutzfeldt Jakob Syndrome"[Title/Abstract]) OR "Syndrome, Creutzfeldt-Jakob"[Title/Abstract]) OR "Creutzfeldt-Jakob Disease"[Title/Abstract]) OR "Creutzfeldt Jakob Disease"[Title/Abstract]) OR "Disease, Creutzfeldt-Jakob"[Title/Abstract]) OR "Jakob-Creutzfeldt Disease"[Title/Abstract]) OR "Disease, Jakob-Creutzfeldt"[Title/Abstract]) OR "Jakob Creutzfeldt Disease"[Title/Abstract]) OR "CJD (Creutzfeldt-Jakob Disease)"[Title/Abstract]) OR "CJD (Creutzfeldt Jakob Disease)"[Title/Abstract]) OR "Spongiform Encephalopathy, Subacute"[Title/Abstract]) OR "Encephalopathies, Subacute Spongiform"[Title/Abstract]) OR "Encephalopathy, Subacute Spongiform"[Title/Abstract]) OR "Spongiform Encephalopathies, Subacute"[Title/Abstract]) OR "Subacute Spongiform Encephalopathies"[Title/Abstract]) OR "Subacute Spongiform Encephalopathy"[Title/Abstract]) OR "Creutzfeldt Jacob Disease"[Title/Abstract]) OR "Variant Creutzfeldt Jakob Disease"[Title/Abstract]) OR "Jacob Disease, Creutzfeldt"[Title/Abstract]) OR "Jakob-Creutzfeldt Syndrome"[Title/Abstract]) OR "Jakob Creutzfeldt Syndrome"[Title/Abstract]) OR "Syndrome, Jakob-Creutzfeldt"[Title/Abstract]) OR "Creutzfeldt-Jakob Disease, Familial"[Title/Abstract]) OR "Creutzfeldt Jakob Disease, Familial"[Title/Abstract]) OR "Creutzfeldt-Jakob Diseases, Familial"[Title/Abstract]) OR "Disease, Familial Creutzfeldt-Jakob"[Title/Abstract]) OR "Familial Creutzfeldt-Jakob Diseases"[Title/Abstract]) OR "Familial Creutzfeldt-Jakob Disease"[Title/Abstract]) OR "Familial Creutzfeldt Jakob Disease"[Title/Abstract]) OR "New Variant Creutzfeldt-Jakob Disease"[Title/Abstract]) OR "New Variant Creutzfeldt Jakob Disease"[Title/Abstract]) OR "Creutzfeldt-Jakob Disease, Variant"[Title/Abstract]) OR "Creutzfeldt Jakob Disease, Variant"[Title/Abstract]) OR "Creutzfeldt-Jakob Disease, New Variant"[Title/Abstract]) OR "Creutzfeldt Jakob Disease, New Variant"[Title/Abstract]) OR "Variant Creutzfeldt-Jakob Disease"[Title/Abstract]) OR "V CJD (Variant Creutzfeldt Jakob Disease)"[Title/Abstract]) OR "V-CJD (Variant-Creutzfeldt-Jakob Disease)"[Title/Abstract] OR ((((((((((((((((((((((((("Huntington Disease"[MeSH Terms]) OR "Huntington Disease"[Title/Abstract]) OR "Akinetic Rigid Variant of Huntington Disease"[Title/Abstract]) OR "Huntington Chorea"[Title/Abstract]) OR "Chorea, Huntington"[Title/Abstract]) OR "Huntington's Disease"[Title/Abstract]) OR "chronic progressive hereditary chorea (huntington)"[Title/Abstract]) OR "Huntington Chronic Progressive Hereditary Chorea"[Title/Abstract]) OR "progressive chorea, chronic hereditary (huntington)"[Title/Abstract]) OR "progressive chorea, hereditary, chronic (huntington)"[Title/Abstract]) OR "Huntington's Chorea"[Title/Abstract]) OR "Chorea, Huntington's"[Title/Abstract]) OR "chorea, chronic progressive/hereditary (huntington)"[Title/Abstract]) OR "Huntington Disease, Late Onset"[Title/Abstract]) OR "Late-Onset Huntington Disease"[Title/Abstract]) OR "Huntington Disease, Late-Onset"[Title/Abstract]) OR "Late Onset Huntington Disease"[Title/Abstract]) OR "Juvenile Huntington Disease"[Title/Abstract]) OR "Juvenile-Onset Huntington Disease"[Title/Abstract]) OR "Juvenile Onset Huntington Disease"[Title/Abstract]) OR "Huntington Disease, Juvenile-Onset"[Title/Abstract]) OR "Huntington Disease, Juvenile Onset"[Title/Abstract]) OR "Huntington Disease, Juvenile"[Title/Abstract]) OR "Akinetic-Rigid Variant of Huntington Disease"[Title/Abstract]) OR "Huntington Disease, Akinetic Rigid Variant"[Title/Abstract]) OR "Huntington Disease, Akinetic-Rigid Variant"[Title/Abstract] OR  ((((((((((((((((((((((((((((((((((((((((((((((((((((((((((((((((((((((((((((((((((((((((((((((((((((((((((((((((((((((((((((((((("Neurodegenerative Diseases"[MeSH Terms]) OR "Neurodegenerative Diseases"[Title/Abstract]) OR "Degenerative Diseases, Nervous System"[Title/Abstract]) OR "Neurodegenerative Disease"[Title/Abstract]) OR "Degenerative Diseases, Neurologic"[Title/Abstract]) OR "Neurologic Degenerative Disease"[Title/Abstract]) OR "Degenerative Neurologic Diseases"[Title/Abstract]) OR "Degenerative Neurologic Disease"[Title/Abstract]) OR "Neurologic Disease, Degenerative"[Title/Abstract]) OR "Neurologic Diseases, Degenerative"[Title/Abstract]) OR "Nervous System Degenerative Diseases"[Title/Abstract]) OR "Neurodegenerative Disorders"[Title/Abstract]) OR "Neurodegenerative Disorder"[Title/Abstract]) OR "Neurologic Degenerative Conditions"[Title/Abstract]) OR "Degenerative Condition, Neurologic"[Title/Abstract]) OR "Degenerative Conditions, Neurologic"[Title/Abstract]) OR "Neurologic Degenerative Condition"[Title/Abstract]) OR "Neurologic Degenerative Diseases"[Title/Abstract]) OR "Degenerative Diseases, Central Nervous System"[Title/Abstract]) OR "Degenerative Neurologic Disorders"[Title/Abstract]) OR "Degenerative Neurologic Disorder"[Title/Abstract]) OR "Neurologic Disorder, Degenerative"[Title/Abstract]) OR "Neurologic Disorders, Degenerative"[Title/Abstract]) OR "Degenerative Diseases, Spinal Cord"[Title/Abstract]) OR "Parkinson Disease"[MeSH Terms]) OR "Parkinson Disease"[Title/Abstract]) OR "Paralysis Agitans"[Title/Abstract]) OR "Idiopathic Parkinson's Disease"[Title/Abstract]) OR "Lewy Body Parkinson Disease"[Title/Abstract]) OR "Lewy Body Parkinson's Disease"[Title/Abstract]) OR "Primary Parkinsonism"[Title/Abstract]) OR "Parkinsonism, Primary"[Title/Abstract]) OR "Parkinson Disease, Idiopathic"[Title/Abstract]) OR "Parkinson's Disease"[Title/Abstract]) OR "Parkinson's Disease, Idiopathic"[Title/Abstract]) OR "Parkinson's Disease, Lewy Body"[Title/Abstract]) OR "Idiopathic Parkinson Disease"[Title/Abstract]) OR "Alzheimer Disease"[MeSH Terms]) OR "Alzheimer Disease"[Title/Abstract]) OR "Disease, Alzheimer's"[Title/Abstract]) OR "Disease, Alzheimer"[Title/Abstract]) OR "Alzheimer Sclerosis"[Title/Abstract]) OR "Sclerosis, Alzheimer"[Title/Abstract]) OR "Alzheimer Syndrome"[Title/Abstract]) OR "Syndrome, Alzheimer"[Title/Abstract]) OR "Alzheimer Dementia (AD)"[Title/Abstract]) OR "Dementia, Alzheimer (AD)"[Title/Abstract]) OR "Alzheimer-Type Dementia (ATD)"[Title/Abstract]) OR "Alzheimer Type Dementia (ATD)"[Title/Abstract]) OR "Dementia, Alzheimer-Type (ATD)"[Title/Abstract]) OR "Primary Senile Degenerative Dementia"[Title/Abstract]) OR "Dementia, Senile"[Title/Abstract]) OR "Senile Dementia"[Title/Abstract]) OR "Dementia, Alzheimer Type"[Title/Abstract]) OR "Alzheimer Type Dementia"[Title/Abstract]) OR "Senile Dementia, Alzheimer Type"[Title/Abstract]) OR "Alzheimer Type Senile Dementia"[Title/Abstract]) OR "Dementia, Primary Senile Degenerative"[Title/Abstract]) OR "Alzheimer's Disease"[Title/Abstract]) OR "Presenile Alzheimer Dementia*"[Title/Abstract]) OR "Acute Confusional Senile Dementia"[Title/Abstract]) OR "Senile Dementia, Acute Confusional"[Title/Abstract]) OR "Dementia, Presenile"[Title/Abstract]) OR "Presenile Dementia"[Title/Abstract]) OR "Alzheimer Disease, Late Onset"[Title/Abstract]) OR "Late Onset Alzheimer Disease"[Title/Abstract]) OR "Alzheimer's Disease, Focal Onset"[Title/Abstract]) OR "Focal Onset Alzheimer's Disease"[Title/Abstract]) OR "Familial Alzheimer Disease (FAD)"[Title/Abstract]) OR "Alzheimer Disease, Early Onset"[Title/Abstract]) OR "Early Onset Alzheimer Disease"[Title/Abstract]) OR "multiple sclerosis"[MeSH Terms]) OR "multiple sclerosis"[Title/Abstract]) OR "Multiple Sclerosis, Acute Fulminating"[Title/Abstract]) OR "Sclerosis, Multiple"[Title/Abstract]) OR "Sclerosis, Disseminated"[Title/Abstract]) OR "Disseminated Sclerosis"[Title/Abstract]) OR "MS (Multiple Sclerosis)"[Title/Abstract]) OR "Pick Disease of the Brain"[MeSH Terms]) OR "Pick Disease of the Brain"[Title/Abstract]) OR "Circumscribed Lobar Atrophy of the Brain"[Title/Abstract]) OR "Brain Atrophy, Circumscribed Lobar"[Title/Abstract]) OR "Pick Disease"[Title/Abstract]) OR "Disease, Pick"[Title/Abstract]) OR "Picks Disease of Brain"[Title/Abstract]) OR "Lobar Atrophy Of Brain"[Title/Abstract]) OR "Pick's Disease"[Title/Abstract]) OR "Disease, Pick's"[Title/Abstract]) OR "Picks Disease"[Title/Abstract]) OR "Lobar Atrophy of the Brain"[Title/Abstract]) OR "Dementia with Lobar Atrophy"[Title/Abstract]) OR "Neuronal Cytoplasmic Inclusions"[Title/Abstract]) OR "Pick Disease Of Brain"[Title/Abstract]) OR "Lobar Atrophy (Brain)"[Title/Abstract]) OR "Atrophies, Lobar (Brain)"[Title/Abstract]) OR "Atrophy, Lobar (Brain)"[Title/Abstract]) OR "Lobar Atrophies (Brain)"[Title/Abstract]) OR "Dementia, Vascular"[MeSH Terms]) OR "Dementia, Vascular"[Title/Abstract]) OR "Binswanger Encephalopathy"[Title/Abstract]) OR "Dementias, Vascular"[Title/Abstract]) OR "Vascular Dementias"[Title/Abstract]) OR "Vascular Dementia"[Title/Abstract]) OR "Vascular Dementia, Acute Onset"[Title/Abstract]) OR "Acute Onset Vascular Dementia"[Title/Abstract]) OR "Subcortical Vascular Dementia"[Title/Abstract]) OR "Dementia, Subcortical Vascular"[Title/Abstract]) OR "Dementias, Subcortical Vascular"[Title/Abstract]) OR "Subcortical Vascular Dementias"[Title/Abstract]) OR "Vascular Dementia, Subcortical"[Title/Abstract]) OR "Vascular Dementias, Subcortical"[Title/Abstract]) OR "Arteriosclerotic Dementia"[Title/Abstract]) OR "Arteriosclerotic Dementias"[Title/Abstract]) OR "Dementia, Arteriosclerotic"[Title/Abstract]) OR "Dementias, Arteriosclerotic"[Title/Abstract]) OR "Binswanger Disease"[Title/Abstract]) OR "Disease, Binswanger"[Title/Abstract]) OR "Chronic Progressive Subcortical Encephalopathy"[Title/Abstract]) OR "Encephalopathies, Subcortical Arteriosclerotic"[Title/Abstract]) OR "Leukoencephalopathy, Subcortical"[Title/Abstract]) OR "Leukoencephalopathies, Subcortical"[Title/Abstract]) OR "Subcortical Leukoencephalopathies"[Title/Abstract]) OR "Encephalopathy, Subcortical Arteriosclerotic"[Title/Abstract]) OR "Binswanger's Disease"[Title/Abstract]) OR "Binswangers Disease"[Title/Abstract]) OR "Disease, Binswanger's"[Title/Abstract]) OR "Encephalopathy, Subcortical, Chronic Progressive"[Title/Abstract]) OR "Subcortical Encephalopathy, Chronic Progressive"[Title/Abstract]) OR "Subcortical Leukoencephalopathy"[Title/Abstract]) OR "Subcortical Arteriosclerotic"[Title/Abstract] |
| Scopus | ( ( ( TITLE-ABS-KEY ( "creutzfeldt syndrome" ) OR TITLE-ABS-KEY ( "Jakob Creutzfeldt Disease" ) OR TITLE-ABS-KEY ( "Subacute Spongiform Encephalopath*" ) OR TITLE-ABS-KEY ( "Familial Creutzfeldt Jakob Disease*" ) OR TITLE-ABS-KEY ( "New Variant Creutzfeldt Jakob Disease" ) OR TITLE-ABS-KEY ( "Variant Creutzfeldt Jakob Disease" ) OR TITLE-ABS-KEY ( "Huntington* Disease" ) OR TITLE-ABS-KEY ( "Huntington* Chorea" ) OR TITLE-ABS-KEY ( "Chronic Progressive Hereditary Chorea" ) OR TITLE-ABS-KEY ( "latent huntington disease" ) OR TITLE-ABS-KEY ( "Juvenile Huntington Disease" ) OR TITLE-ABS-KEY ( "Juvenile Onset Huntington Disease" ) OR TITLE-ABS-KEY ( "akinetic rigid variant off huntington disease" ) OR TITLE-ABS-KEY ( "Motor Neuron Disease*" ) OR TITLE-ABS-KEY ( "Motor System Disease*" ) OR TITLE-ABS-KEY ( "Familial Motor Neuron Disease" ) OR TITLE-ABS-KEY ( "Lateral Scleros*" ) OR TITLE-ABS-KEY ( "Primary Lateral Scleros*" ) OR TITLE-ABS-KEY ( "Upper Motor Neuron Disease" ) OR TITLE-ABS-KEY ( "Secondary Motor Neuron Disease" ) OR TITLE-ABS-KEY ( "Anterior Horn Cell Disease" ) ) ) OR ( ( TITLE-ABS-KEY ( "multiple sclerosis" ) OR TITLE-ABS-KEY ( "Disseminated Sclerosis" ) OR TITLE-ABS-KEY ( "Multiple Sclerosis, Acute Fulminating" ) OR TITLE-ABS-KEY ( "pick disease off the brain" ) OR TITLE-ABS-KEY ( "Pick* Disease" ) OR TITLE-ABS-KEY ( "lobar atrophy off brain" ) OR TITLE-ABS-KEY ( "Dementia with Lobar Atrophy and Neuronal Cytoplasmic Inclusions" ) OR TITLE-ABS-KEY ( "circumscribed lobar atrophy off the brain" ) OR TITLE-ABS-KEY ( "Dementia*, Vascular" ) OR TITLE-ABS-KEY ( "Acute Onset Vascular Dementia" ) OR TITLE-ABS-KEY ( "Subcortical Vascular Dementia*" ) OR TITLE-ABS-KEY ( "Arteriosclerotic Dementia*" ) OR TITLE-ABS-KEY ( "Binswanger Disease" ) OR TITLE-ABS-KEY ( "Chronic Progressive Subcortical Encephalopathy" ) OR TITLE-ABS-KEY ( "Binswanger* Encephalopathy" ) OR TITLE-ABS-KEY ( "Subcortical Leukoencephalopath*" ) OR TITLE-ABS-KEY ( "Encephalopathy, Subcortical Arteriosclerotic" ) OR TITLE-ABS-KEY ( "Subcortical Encephalopathy, Chronic Progressive" ) OR TITLE-ABS-KEY ( "Binswanger's Encephalopathy" ) ) ) OR ( ( TITLE-ABS-KEY ( "Neurodegenerative Disease*" ) OR TITLE-ABS-KEY ( "Neurologic Degenerative Disease*" ) OR TITLE-ABS-KEY ( "Neurodegenerative Disorder*" ) OR TITLE-ABS-KEY ( "Neurologic Degenerative Condition*" ) OR TITLE-ABS-KEY ( "Degenerative Neurologic Disorder*" ) OR TITLE-ABS-KEY ( "Idiopathic Parkinson* Disease" ) OR TITLE-ABS-KEY ( "Lewy Body Parkinson* Disease" ) OR TITLE-ABS-KEY ( "Primary Parkinsonism" ) OR TITLE-ABS-KEY ( "Paralysis Agitans" ) OR TITLE-ABS-KEY ( "Parkinson* Disease" ) OR TITLE-ABS-KEY ( "Alzheimer* Disease" ) OR TITLE-ABS-KEY ( "Alzheimer Sclerosis" ) OR TITLE-ABS-KEY ( "Alzheimer Syndrome" ) OR TITLE-ABS-KEY ( "Alzheimer Dementia" ) OR TITLE-ABS-KEY ( "Alzheimer Type Dementia" ) OR TITLE-ABS-KEY ( "Primary Senile Degenerative Dementia" ) OR TITLE-ABS-KEY ( "Senile Dementia" ) OR TITLE-ABS-KEY ( "Degenerative Diseases, Spinal Cord" ) OR TITLE-ABS-KEY ( "Degenerative Diseases, Central Nervous System" ) OR TITLE-ABS-KEY ( "Alzheimer Type Senile Dementia" ) OR TITLE-ABS-KEY ( "Acute Confusional Senile Dementia" ) OR TITLE-ABS-KEY ( "Presenile Dementia" ) OR TITLE-ABS-KEY ( "Late Onset Alzheimer Disease" ) OR TITLE-ABS-KEY ( "Focal Onset Alzheimer's Disease" ) ) ) ) AND ( ( TITLE-ABS-KEY ( "Chronic Periodontitis" ) OR TITLE-ABS-KEY ( "chronic periodontitis" ) OR TITLE-ABS-KEY ( "Adult Periodontitis" ) OR TITLE-ABS-KEY ( "adult periodontitis" ) OR TITLE-ABS-KEY ( "Periodontal Atroph*" ) OR TITLE-ABS-KEY ( "atrophy off periodontium" ) OR TITLE-ABS-KEY ( "Periodontium Atroph*" ) OR TITLE-ABS-KEY ( "Gingivo Osseous Atroph*" ) OR TITLE-ABS-KEY ( "Alveolar Bone Loss*" ) OR TITLE-ABS-KEY ( "Alveolar Process Atroph*" ) OR TITLE-ABS-KEY ( "Alveolar Resorption*" ) OR TITLE-ABS-KEY ( "Periodontal Bone Loss*" ) OR TITLE-ABS-KEY ( "Periodontal Resorption*" ) OR TITLE-ABS-KEY ( "Alveolar Bone Atroph*" ) ) ) |
| WEB OF SCIENCE | Tópico: (Creutzfeldt-Jakob Syndrome) OR Tópico: (Jakob Creutzfeldt Disease) OR Tópico: (Subacute Spongiform Encephalopath*) OR Tópico: (Familial Creutzfeldt Jakob Disease*) OR Tópico: (New Variant Creutzfeldt Jakob Disease) OR Tópico: (Variant Creutzfeldt Jakob Disease) OR Tópico: (Huntington* Disease) OR Tópico: (Huntington* Chorea) OR Tópico: (Chronic Progressive Hereditary Chorea (Huntington)) OR Tópico: (Late-Onset Huntington Disease) OR Tópico: (Juvenile Huntington Disease) OR Tópico: (Juvenile Onset Huntington Disease) OR Tópico: (Akinetic Rigid Variant of Huntington Disease) OR Tópico: (Motor Neuron Disease*) OR Tópico: (Motor System Disease*) OR Tópico: (Familial Motor Neuron Disease) OR Tópico: (Lateral Scleros*) OR Tópico: (Primary Lateral Scleros*) OR Tópico: (Upper Motor Neuron Disease) ORTópico: (Secondary Motor Neuron Disease) OR Tópico: (Anterior Horn Cell Disease) OR Tópico: (multiple sclerosis) OR Tópico: (Disseminated Sclerosis) OR Tópico: (Multiple Sclerosis, Acute Fulminating) OR Tópico: (Pick Disease of the Brain) OR Tópico: (Pick* Disease) OR Tópico: (Lobar Atrophy Of Brain) OR Tópico: (Dementia with Lobar Atrophy and Neuronal Cytoplasmic Inclusions) OR Tópico: (Circumscribed Lobar Atrophy of the Brain) OR Tópico: (Dementia*, Vascular) OR Tópico: (Acute Onset Vascular Dementia) OR Tópico: (Subcortical Vascular Dementia*) OR Tópico: (Arteriosclerotic Dementia*) ORTópico: (Binswanger Disease) OR Tópico: (Chronic Progressive Subcortical Encephalopathy) OR Tópico: (Binswanger* Encephalopathy) OR Tópico: (Subcortical Leukoencephalopath*) OR Tópico: (Encephalopathy, Subcortical Arteriosclerotic) OR Tópico: (Subcortical Encephalopathy, Chronic Progressive) OR Tópico: (Binswanger's Encephalopathy) OR Tópico: (Neurodegenerative Disease*) OR Tópico: (Neurologic Degenerative Disease*) OR Tópico: (Nervous System Degenerative Diseases) OR Tópico: (Neurodegenerative Disorder*) OR Tópico: (Neurologic Degenerative Condition*) OR Tópico: (Degenerative Neurologic Disorder*) OR Tópico: (Degenerative Diseases, Spinal Cord) OR Tópico: (Degenerative Diseases, Central Nervous System) OR Tópico: (Idiopathic Parkinson* Disease) OR Tópico: (Lewy Body Parkinson* Disease) OR Tópico: (Primary Parkinsonism) OR Tópico: (Paralysis Agitans) OR Tópico: (Parkinson* Disease) OR Tópico: (Alzheimer* Disease) OR Tópico: (Alzheimer Sclerosis) OR Tópico: (Alzheimer Syndrome) OR Tópico: (Alzheimer Dementia (AD)) OR Tópico: (Alzheimer Type Dementia) OR Tópico: (Primary Senile Degenerative Dementia) OR Tópico: (Senile Dementia) ORTópico: (Alzheimer Type Senile Dementia) OR Tópico: (Acute Confusional Senile Dementia) OR Tópico: (Presenile Dementia) OR Tópico: (Late Onset Alzheimer Disease) OR Tópico: (Focal Onset Alzheimer's Disease) AND Tópico: (Chronic Periodontitis) OR Tópico: (Chronic Periodontitides) OR Tópico: (Adult Periodontitis) ORTópico: (Adult Periodontitides) OR Tópico: (Periodontal Atroph*) OR Tópico: (Atrophy of Periodontium) ORTópico: (Periodontium Atroph*) OR Tópico: (Gingivo Osseous Atroph*) OR Tópico: (Alveolar Bone Loss*) ORTópico: (Alveolar Process Atroph*) OR Tópico: (Alveolar Resorption*) OR Tópico: (Periodontal Bone Loss*) OR Tópico: (Periodontal Resorption*) OR Tópico: (Alveolar Bone Atroph*). |
| OPEN GREY | Neurodegenerative diseases or Alzheimer Disease or Parkinson Disease or Huntington Disease or Amyotrophic Lateral Sclerosis or multiple sclerosis and Periodontitis. |

**Supplementary Table 2.** Domains and Risk of Bias considered in Risk of Bias evaluation according to Fowkes and Fulton (Fowkes and Fulton, 1991), adapted Almeida et al (Almeida et al., 2017).

| Guideline | Checklist | Description |
| --- | --- | --- |
| Study design apropriate to objectives? | Objective common design | The type of study was marked in the appropriate type of study. If the type of study was appropriate according to the study design was marked as "0" and as "++" if it was not appropriate. |
|  | Prevalence Cross-sectional |  |
|  | Prognosis Cohort |  |
|  | Treatment Controled trial |  |
|  | Cause Cohort, case-control, cross-sectional |  |
| Study sample representative? | Source of sample | The domain was considered (0) in cases of detailed origin, (+) to specified origin of only one group and (++) in cases of absence of specification of the origin of the groups |
|  | Sampling method | The item was assigned (0) for full description of sampling method, (+) for poor or no description of sample method, with no problem in matching between groups and (++) for poor or no description of sample method, interfering in matching of the groups |
|  | Sample size | A minor problem (+) was considered when the sample was not representative or did not report a sample calculation. To a major problem, (++) was considered when no sample calculation was provided and the number of participants was less than 50 participants, (0) was considered in absence of the above factors. |
|  | Entry criteria/exclusion | A minor (+) problem was attributed when the control group was a case of a history of previous neurodegenerative diseases, dementia or cognitive impairment. In the case of more than one item previously mentioned, considered a major problem (++). |
|  | Non-respondents | The (0) was attributed when there was no refusal to participation in the study, (+) was assigned when there was refusal, but did not compromise the sample, and (++) when there was refusal and impairment of the sample size. |
| Control group acceptable? | Definition of controls | It was attributed (0) when all characteristics of control group were described, (+) when any information was pendent as the origin of control group, the selection criterions and a different origin between case and control groups and (++) when two or more items described in previously items. |
|  | Source of controls | It was considered (0) when control group was referred, (+) when the origin of groups was different, but with reasons and (++) when the groups present different origins without reasons. |
|  | Matching/randomization | In this item, (0) was assigned to cases of randomized/matched groups, (+) to cases of no description of randomization, but with matching of groups and (++) to no description of randomization or matching. |
|  | Comparable characteristics | It was attributed (0) to matched groups or not matched by the impossibility of being subsequently adjusted and (++) presence of unpaired variables that were not paired or adjusted. |
| Quality of measurements and outcomes? | Validity | It was considered (0) when the evaluation method applied is appropriate; (+) when using a single method, but with appropriate sensitivity with good specificity; (++) when using a single method, without an adequate specificity or good sensitivity. |
|  | Reproducibility | It was considered (0) whether the evaluation methods were well described; (+) when a lack description of any step of the method was presented, for example, the identification of the patients of the groups studied in laboratory samples, evaluations at different times or application of different methods between groups of certain pathology; (++) when two or more of the previous items are present. |
|  | Blindness | The condition of the study participants was considered to be "Blind", in this case being assigned the signal (0), in cases of "not blind" the signal (++) was attributed |
|  | Quality control | It was considered a problem when the evaluators were not calibrated; when ungraduated students carried out the assessment without supervision of a qualified dentist; analysis of periodontitis only radiographic and/or depth of periodontal pockets, to evaluate less than three dental faces or not to mention how many faces were evaluated. When two of these problems were identified, it was considered as a minor problem (+) and major problems (++) if more than two of these characteristics were described. |
| Completeness | Compliance | It was assigned (0) for a sample size that remains the same from the beginning to the end or decreases without compromising the power of the test; (+) for differences in sample size at the end of the study, compromising the power of the test, but with reasons and adjusts; (++) for difference in sample size at the end of the study, compromising the power of the test, without reasons. |
|  | Drop outs | The (0) was scored when there is no loss during the study, (+) when there is withdrawal that involves the inclusion criteria, such as age, sex, (++) when there is withdrawal and it compromises more than one criterion. |
|  | Deaths | This item was scored as Not Applicable (NA). |
|  | Missing data | In this item, (0) was assigned to cases of randomized/matched groups, (+) to cases of no description of randomization, but with matching of groups and (++) to no description of randomization or matching. |
| Distorting influences? | Extraneous treatments | In this item, (0) was considered when there were no external influences; (+) when there are external influences, but that does not interfere in the results; (++) when there are external influences and interferes with the results. |
|  | Contamination | This item was scored as Not Applicable (NA). |
|  | Changes over time | In this item, (0) was attributed to data collected in the same time period; (+) to data collected from the control group and the study group at different times that may cause distortions; (++) when the previous item was associated with data from studies already published. |
|  | Confounding factors | A problem was considered in case of the presence of men and women under the age of 45, menopausal, smoker, diabetic and obese women. A "minor" (+) problem was attributed when 1 or 2 of these characteristics were present and a "larger" (++) problem if there were 3 or more. |
|  | Distortion reduced by analysis | It was considered (0) when it cites the adjustments of the covariates that present distortions; (+) when the article report adjustment but does not say the criteria; (++) when a distortion was identified, without adjustment. |
| Summary questions | **Bias:** | “YES” or "NO" answers were assigned for each question. If the answer is NO at the three questions, the article is considered reliable, with low risk of bias. |
|  | Are the results erroneously biased in certain direction? |  |
|  | **Confounding:** |  |
|  | Are there any serious confusing or other distoring influences? |  |
|  | **Chance:** |  |
|  | Is it likely that the results ocurred by chance? |  |

**References**

Almeida, A., Fagundes, N.C.F., Maia, L.C., and Lima, R.R. (2017). Is There An Association Between Periodontitis And Atherosclerosis In Adults? A Systematic Review. *Curr Vasc Pharmacol*.

Fowkes, F.G., and Fulton, P.M. (1991). Critical appraisal of published research: introductory guidelines. *BMJ : British Medical Journal* 302**,** 1136-1140.

Moher, D., Liberati, A., Tetzlaff, J., and Altman, D.G. (2009). Preferred reporting items for systematic reviews and meta-analyses: the PRISMA statement. *PLoS Med* 6**,** e1000097.

**Supplementary Table 3.** Reasons for exclusion

| **Reference** | **Reason for exclusion** |
| --- | --- |
| Chen, C. K., Huang, J. Y., Wu, Y. T., & Chang, Y. C. (2018). Dental scaling decreases the risk of Parkinson’s disease: a nationwide population-based nested case-control study. International journal of environmental research and public health, 15(8), 1587. | Analysis method unable to assess periodontitis accordingly our eligibility criteria |
| Choi, S., Kim, K., Chang, J., Kim, S. M., Kim, S. J., Cho, H. J., & Park, S. M. (2019). Association of chronic periodontitis on Alzheimer's disease or vascular dementia. Journal of the American Geriatrics Society, 67(6), 1234-1239. | Absence of control group. |
| Chen, C. K., Wu, Y. T., & Chang, Y. C. (2017). Association between chronic periodontitis and the risk of Alzheimer’s disease: a retrospective, population-based, matched-cohort study. Alzheimer's Research & Therapy, 9(1), 56. | Absence of control group. |
| Dulamea, A. O., Boscaiu, V., & Sava, M. M. (2015). Disability status and dental pathology in multiple sclerosis patients. Multiple sclerosis and related disorders, 4(6), 567-571. | Absence of control group. |
| Farhad, S. Z., Amini, S., Khalilian, A., Barekatain, M., Mafi, M., Barekatain, M., & Rafei, E. (2014). The effect of chronic periodontitis on serum levels of tumor necrosis factor-alpha in Alzheimer disease. Dental research journal, 11(5), 549. | Absence of control group. |
| Holmer, J., Eriksdotter, M., Schultzberg, M., Pussinen, P. J., & Buhlin, K. (2018). Association between periodontitis and risk of Alzheimer′ s disease, mild cognitive impairment and subjective cognitive decline: A case–control study. Journal of Clinical Periodontology, 45(11), 1287-1298. | Analysis method unable to assess periodontitis accordingly our eligibility criteria |
| Nakayama, R., Nishiyama, A., Matsuda, C., Nakayama, Y., Hakuta, C., & Shimada, M. (2018). Oral health status of hospitalized amyotrophic lateral sclerosis patients: a single-centre observational study. Acta Odontologica Scandinavica, 76(4), 294-298. | Absence of control group. |
| Sochocka, M., Sender-Janeczek, A., Zwolinska, K., Tomczyk, T., & Leszek, J. (2017). Association between Periodontal Health Status and Cognitive Abilitie s. The Role of Cytokine Profile and Systemic Inflammation. Current Alzheimer Research, 14(9), 978-990. | Absence of control group. |
